# Pyrethroid Treatment of Cattle for Tsetse Control: Reducing Its Impact on Dung Fauna

**DOI:** 10.1371/journal.pntd.0003560

**Published:** 2015-03-04

**Authors:** Glyn A. Vale, John W. Hargrove, Andrew Chamisa, Ian F. Grant, Stephen J. Torr

**Affiliations:** 1 Natural Resources Institute, University of Greenwich, Chatham, United Kingdom; 2 South African Centre for Epidemiological Modelling and Analysis, University of Stellenbosch, Stellenbosch, South Africa; 3 Division of Tsetse Control, Harare, Zimbabwe; 4 Liverpool School of Tropical Medicine, Liverpool, United Kingdom; 5 Warwick Medical School, University of Warwick, Coventry, United Kingdom; Universidad de Buenos Aires, ARGENTINA

## Abstract

**Background:**

African trypansomiases of humans and animals can be controlled by attacking the vectors, various species of tsetse fly. Treatment of cattle with pyrethroids to kill tsetse as they feed is the most cost-effective method. However, such treatments can contaminate cattle dung, thereby killing the fauna which disperse the dung and so play an important role in soil fertility. Hence there is a need to identify cost-effective methods of treating cattle with minimal impact on dung fauna.

**Methodology/Principal Findings:**

We used dung beetles to field bioassay the levels of dung contamination following the use of spray and pour-on formulations of deltamethrin, applied to various parts of the body of cattle in Zimbabwe. Results suggested that dung was contaminated by contact with insecticide on the body surface as the cattle defecated, and by ingestion of insecticide as the cattle licked themselves. Death of dung beetles was reduced to negligible levels by using only the spray and applying it to the legs and belly or legs alone, *i.e*., places where most tsetse feed.

**Conclusion/Significance:**

The restricted applications suitable for minimising the impact on dung fauna have the collateral benefits of improving the economy and convenience of cattle treatments for tsetse control. The demonstration of collateral benefits is one of the surest ways of promoting environmentally friendly procedures.

## Author Summary

In Zimbabwe, we treated cattle with spray or pour-on formulations of deltamethrin, as recommended by the manufacturers for control of tsetse flies. We then gauged the degree of dung contamination, using field bioassays involving adult beetles (Coleoptera: Scarabaeidae). In the first 16 days after whole-body spraying, beetle mortality was up to 18%, as against 76% for pour-on applied to the back or flanks. Specialised treatments involved the application of reduced doses of deltamethrin to only the shoulder or anal area, oral administration and use of a collared animal that could not lick its flank. It seemed that dung was contaminated by contact with insecticide near the anus as the cattle defecated, and by ingestion of insecticide as the cattle licked themselves. Spraying only the legs of cattle produced negligible deaths of beetles when treatment intervals were five or 25 days, but daily treatment caused mortality to rise to ~70% after three weeks. Treating only certain parts of the body surface of cattle reduces the threats to dung fauna and improves the economy and convenience of controlling tsetse.

## Introduction

The tsetse-borne diseases of sleeping sickness in humans and nagana in domestic stock limit health and economic development in much of Africa [1]. An important means of reducing the risk of contracting these diseases is the control of tsetse [1], and the simplest and cheapest method of achieving this is the application of pyrethroids to cattle [2,3]. These insecticides have low mammalian toxicty [4] and they degrade quickly in tropical environments, with half-lives of ~1 week for deltamethrin on vegetation or in soil [5]. However, the cattle treatments can contaminate the dung sufficiently to affect dung fauna, thereby threatening the important role that such fauna play in dung dispersal, and hence soil fertility and the productivity of pastures and crops [6]. For example, in the first week or two after the use of deltamethrin sprays or pour-ons the insecticide can occur at up to 0.1 ppm in the wet weight of freshly dropped dung [7,8]. The survival or reproductive performance of dung fauna can be reduced substantially in dung contaminated with either deltamethrin, cyfluthrin, alphacypermethrin or cypermethrin [7–10]. Modelling suggests that the population impact could be severe, at least with the slower-breeding beetles when the interval of treatment is about a month or less [7,11]. It is regrettable, therefore, that the recommended treatment interval for tsetse control is usually 2–4 weeks [12], not the three months that has sometimes been proposed [13]. Hence, it is necessary to identify strategies that will reduce the environmental impact of short-interval treatments.

One strategy for reducing the impact on dung fauna might be to change the type of insecticide used. For example, flumethrin is less toxic per gram of active ingredient than deltamethrin and some of the other pyrethroids [8–10]. However, the pyrethroids that are least toxic to dung fauna are also less effective against tsetse. Thus, even when flumethrin was applied to cattle at ten times the rate recommended for deltamethrin its performance against tsetse was still relatively poor [13,14]. Another strategy is to reduce the amount of active ingredient that needs to be applied to cattle. This depends partly on the choice of insecticide formulation. For example, using a spray formulation of deltamethrin involves about a third of the active ingredient required for pour-on treatment, and produces only about a third of the level of dung contamination [8]. The amount of active ingredient used can also be reduced by restricting the spray to those individual cattle and the particular body regions on which tsetse feed most, *i.e*, mainly the legs and/or belly [15] of older animals [16].

The exploration of these strategies in Zimbabwe began in January 2000 [8,11] and the preliminary findings [17] suggested that restricted application was a promising means of avoiding risks to dung fauna. Moreover, the restriction seemed to offer the important collateral benefits of reducing insecticide costs by up to 90% [17], making treatment more convenient and ensuring that the tick burden of young stock was not reduced sufficiently to hinder the development of immunity to tick-borne diseases [18]. It was mostly the collateral benefits that captured the interest of veterinarians and livestock owners and which dominated research [15] and field trials, particularly with a view to helping farmers to help themselves [19–23]. Present work in Zimbabwe identified the routes by which pyrethroid enters dung and firmed the indications that contamination can be reduced greatly by restricted application.

## Materials and Methods

### Ethics

All work was performed at Rekomitjie Research Station in the Mana Pools National Park of the Zambezi Valley, Zimbabwe. Staff involved in the experiments were permanent pensionable employees of the Division of Tsetse Control, Government of Zimbabwe and were given regular updates on the purpose and results of the studies. Before recruitment, the Division explains the nature of the work, the risks associated with tsetse, other disease vectors and wild animals, and warns of the social hardships attending life on a remote field station. Recruits sign a document indicating their informed consent to perform the work required. This document is held by the Division. Cattle employed in the work were under regular veterinary supervision. Treatments were given ethical approval by the Division’s Review Committee for Rekomitjie, by Resolution No. 12, in recognition of Licenses 84/80.16.12/9073 (Decatix) and 85/80.16.13/9087 (SpotOn) issued by the Medicines Control Authority of Zimbabwe.

### Insecticides and doses

Deltamethrin was obtained as commercial formulations produced by Cooper (Zimbabwe) Ltd. The formulations, and the manufacturer’s recommended method of application for killing tsetse contacting the whole body surface, were as follows.

1. Decatix—50 g l^-1^ suspension concentrate of deltamethrin, diluted with water to 0.05 g l^-1^ and sprayed to run-off.
2. SpotOn—10 g l^-1^ solution of deltamethrin in oil, as a pour-on applicable as 0.1 ml of formulation per 1 kg of body weight. The standard method of application was to deposit 5 ml on the root of the tail, 5 ml on the top of the head/neck junction, and the remainder along the back. The manufacturer recommended that the insecticide should also be tested when divided into two equal doses, applied in a horizontal line along each flank.

At the recommended rates of application the quantity of deltamethrin deposited per 100 kg of body weight was 0.04 g with Decatix and 0.10 g with SpotOn. Various doses of detlamethrin were also given orally, involving each dose being first mixed with 20 ml of diluent, which was sunflower oil for SpotOn and water for Decatix.

### Cattle and treatments

Test oxen were predominantly Shona cattle, a type of short horned Sanga occurring widely in northern Zimbabwe, and weighed an average of 340 kg (range 160–500). They fed on natural grazings and produced dung that had a mean dry matter content of 16.8% (SD = 2.6, N = 218). Insecticide treatments were given at about 09.00 h, to the oxen restrained in individual crushes. The animals were then grazed separately during the day and held separately at night, in pens with concrete floors and wire-fences that were scrubbed each day. Each of several separate experiments typically involved three types of insecticide treatment and a control that were replicated 3–8 times (mean = 4), with replicates spaced at least one month apart within the wet and cool seasons, *i.e*., December to June, of 2000–2001 and 2012–2013. These are the months in which adult dung beetles appear most abundant in pats at Rekomitjie [8]. Oxen were allocated so that the various replicates of any treatment used different oxen, but so far as possible each ox was given a turn with each treatment.

### Bioassays

Freshly dropped dung from each ox was collected at about 07.00 h and 14.00 h on each day, usually up to 16 days after treatment. One or two artificial pats, each 800 g wet weight, were made from the dung of each ox within one hour of collection and were deployed individually, 10–15 m apart in partly shaded situations in deciduous woodland within 1 km of the station. Bioassays over the 24 h after deployment were performed by the field methods of [8] to assess the percent mortality of adult beetles, mostly Histeridae and Scarabaeinae such as *Aphodius*, *Copris*, *Onitis* and *Sisyphus* spp. which are some of the main dung fauna in Zimbabwe [24] The whole study involved a total of 3408 pats, with a mean of 11.1 beetles per pat (range 0–181).

The percent mortality was taken to be the number of dead and moribund beetles found outside the pats at three inspections up to 24 h after deployment, plus the number of such beetles found inside the pat at the last inspection, all expressed as a percent of all beetles found, *i.e*., the total dead and moribund plus the number alive. The design could thus be regarded as a series of Bernoulli trials, where each dung-beetle death is regarded as a “success”. The data were therefore regarded as binomially distributed, so the 95% confidence limits of the percent mortality in the total samples was calculated using the BinomHigh and BinomLow add-ins of Microsoft Excel. As a check we confirmed that identical limits resulted from analyses carried out using the cii command in Stata (Version 11). Control mortality averaged 0.0% (N = 4760) in the wet months of December to February, and 0.6% (N = 6080) in the drier weather of March to June. Since these figures were low, no correction for control mortality was made.

## Results

Table 1 summarises the overall beetle mortality occurring within the whole 16 days of bioassays performed after each of the main types of treatment in various experiments. However, to understand the mortality more fully it was necessary to examine the data for each day after treatment in each experiment, and to explore variations to the main treatments. The full daily data are given in S1 Spreadsheet, and their indications are extracted below.

**Table 1:** Description and overall impact of each of the main treatments.

Application	Insecticide formulation	Dose mg/kg	Replicates	Different oxen used	Total beetles	Percent dead
Whole body	Decatix	0.40	8	7	5631	7.5
Whole body (back)	SpotOn	1.00	6	6	4126	30.4
Whole body (flank)	SpotOn	1.00	3	3	3188	29.5
Shoulder	SpotOn	0.10–0.15	3	3	1056	1.6
Tail	SpotOn	0.10–0.15	3	3	1074	12.9
Oral	SpotOn	0.10–0.15	3	3	1241	25.5
Legs and belly	Decatix	0.08	3	3	2700	0.7
Legs only	Decatix	0.04	3	3	2793	0.1

Percent of dead beetles among the total collected from dung dropped in 16 days after various formulations of deltamethrin were applied to different body regions at different doses of active ingredient per kg of body mass, in a number of replicates involving different individual oxen.

### Unrestricted application

**Fig 1:**
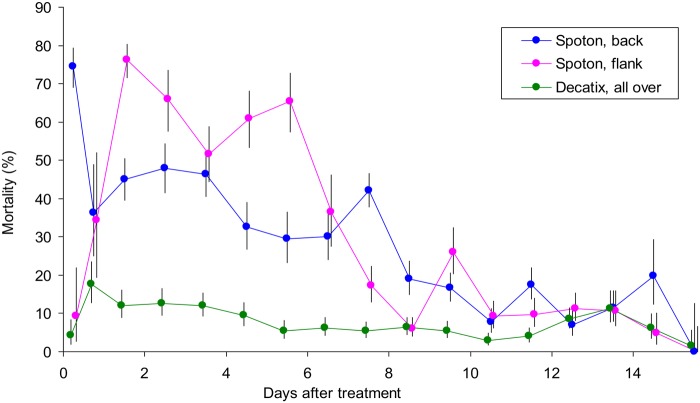
Percent mortality of beetles at pats produced on various days after insecticide applications intended as whole-body treatments. Decatix was sprayed all over and SpotOn was applied to the back or flanks. For the first day after treatment the data for dung produced in the morning and afternoon are separated, but for later days the morning and afternoon data are pooled. Vertical lines through the means indicate the 95% confidence range.

The first experiment employed only those treatments recommended by the manufacturer for effective control of tsetse contacting the whole body surface. The work involved eight replicates with Decatix sprayed all over the body, six with SpotOn deposited on the back and three with SpotOn applied to the flank. Three of the replicates of each treatment were those used previously [8] to show that the bioassay method gives a reliable index of the amount of deltamethrin in the dung. Not surprisingly, therefore, the pooled data of all replicates (Fig. 1) confirmed the earlier indications of chemical and biological assays [8] in showing that the mortality due to SpotOn on the back was high immediately after treatment and declined slowly during the next two weeks. With SpotOn applied to the flanks the mortalities were low immediately after treatment, rising rapidly to high levels in the next week before declining sharply at the start of the second week. Decatix sprayed all over produced relatively low mortalities throughout.

### Route of contamination

The above indication that SpotOn applied to the flanks and back produced distinctive patterns of contamination (Fig. 1) suggested that further variations in application technique could help to elucidate the route by which insecticide enters the dung. For this work the SpotOn dose per animal was 5 ml, *i.e*, about 10–15% of the normal dose that would have been applied to the animals employed. In one treatment the 5 ml was given orally, to simulate licking the insecticide formulation off the coat. In another treatment the 5 ml was applied to the root of the tail, close to where it could contaminate the dung directly. In a third treatment the 5 ml was put on the top front of the shoulder, where it could not be licked. The tail and shoulder positions would each receive about 5 ml during normal treatment with SpotOn on the back.

**Fig 2:**
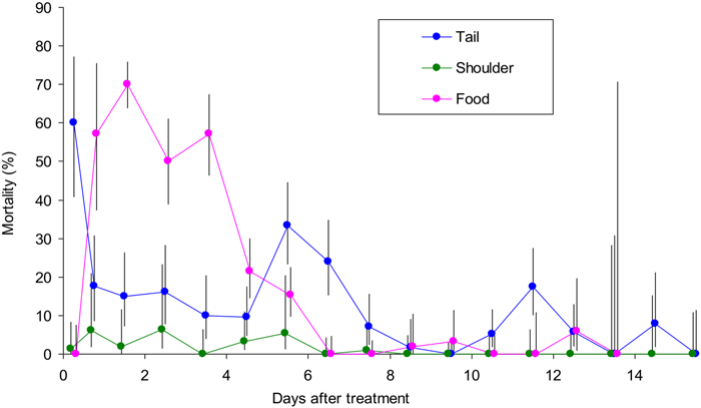
Percent mortality of beetles at pats produced on various days after application of 5 ml of SpotOn in different ways. The dose was applied to the tail or shoulder or given in the food. For the first day after treatment the data for dung produced in the morning and afternoon are separated, but for later days the morning and afternoon data are pooled. Vertical lines through the means indicate the 95% confidence range.

The results from three replicates of each of the three ways of applying the 5 ml of SpotOn (Fig. 2) should be compared with those for the various ways of applying the full dose of SpotOn (Fig. 1). The tail treatment with 5 ml, like the full dose applied to the back, produced a high contamination immediately, consistent with the insecticide being deposited close to where the dung was evacuated and so contacting the dung directly. The oral treatment with 5 ml gave the delayed contamination consistent with the time taken to pass through the gut. The fact that the time course of this contamination was somewhat similar to that of SpotOn applied to the flank (Fig. 1) suggested that much of the contamination due to flank treatment involved licking. The shoulder treatment caused very little contamination, consistent with the insecticide being deposited where it could not quickly contaminate the anal area or be licked. Hence, it seemed that the various levels and timings of mortalities following the back or flank treatments with normal doses of SpotOn (Fig. 1) could be explained by differing extents to which the treatments favoured the oral and anal routes of contamination.

The importance of licking after the flank application of a full dose of SpotOn was investigated further using one animal of 440 kg, *i.e*, with 22 ml of the pour-on applied on each side. The animal was prevented from licking its flanks by being given a foam-rubber collar that extended outwards for 60 cm from the neck immediately behind the head. Dung produced in the first 6 h after treatment was ignored, since in that time licking could have had little effect (Fig. 1). In the remainder of the first week after treatment, when licking was suspected of having its greatest potential effect (Fig. 1), the collared animal gave a beetle mortality of 31% (CL = 22–41%, N = 91), which was half of the 62% (CL = 59–65%, N = 1141) for non-collared animals (Fig. 1). At 8–15 days after treatment, when much of the insecticide would have spread all over the body [12,25], the collared ox gave a beetle mortality of 17% (CL = 9–27%, N = 78), *i.e*., not significantly different from the 11% (CL = 10–13%, N = 1994) with non-collared oxen (Fig. 1). The results are consistent with the idea that licking is responsible for much or most of the dung contamination associated with flank treatment, especially the very high contamination occurring a few days after such treatment.

### Various oral doses

**Fig 3:**
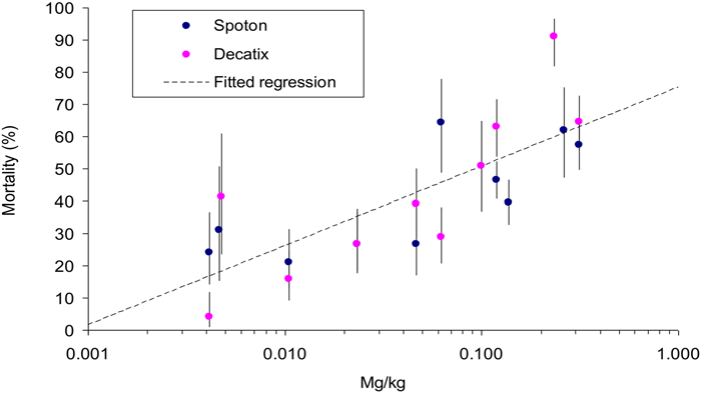
Percent mortality of beetles at pats in the first week after oral administration of various doses of deltamethrin. The deltamethrin was given as SpotOn or Decatix, at doses measured as mg of active ingredient per kg of body mass. Vertical lines through the plots indicate the 95% confidence range. The regression line is fitted to the pooled data for SpotOn and Decatix.

The importance of licking was investigated further by giving oral doses of 5 ml, 1 ml and 0.2 ml of SpotOn. The quantities of deltamethrin in these doses, *i.e*, 25, 5 and 1 mg of active ingredient, were also given orally as Decatix. Each dose of Decatix or SpotOn was converted to doses of deltamethrin per kg of body mass. Virtually all of the beetle mortality resulting from these oral treatments occurred between six hours and a week after treatment, as exemplified in Fig. 2. Hence all results were expressed as the mean mortality in pats dropped in that period. This mortality seemed linearly related to the log of dose (Fig. 3), with no significant difference in the regressions for Decatix and SpotOn. The pooled data for both formulations indicated a significant (P<0.001) increase in mortality with increased dose. The results offer additional evidence that the oral route of contamination can be important.

### Restricted application to legs and belly

**Fig 4:**
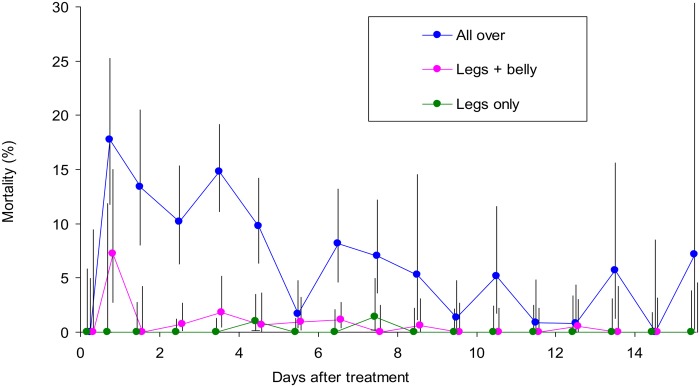
Percent mortality of beetles at pats on various days after application of Decatix in different ways. The Decatix was sprayed all over the body or onto only the legs and belly or legs only. For the first day after treatment the data for dung produced in the morning and afternoon are separated, but for later days the morning and afternoon data are pooled. Vertical lines through the means indicate the 95% confidence range.

Restricted applications of Decatix spray to the legs and belly, and to the legs alone, were compared with Decatix sprayed over the whole body, in an experiment involving three replicates of each treatment. The results (Fig. 4) showed that mortalities associated with the restricted applications were very low, averaging 0.1% for the legs only treatment and 0.7% for the legs plus belly treatment, as against 7.7% for the whole body application.

**Fig 5:**
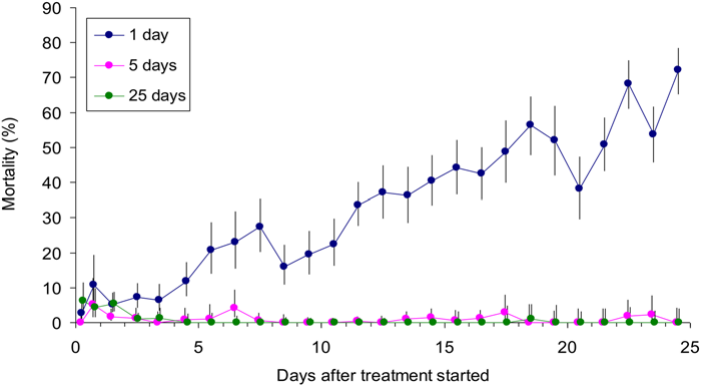
Percent mortality of beetles at pats produced on various days after the start of different regimes of Decatix treatment to the legs. The regimes involved spraying the Decatix at intervals of one, five and 25 days. For the first day of each regime the data for dung produced in the morning and afternoon are separated, but for later days the morning and afternoon data are pooled. Vertical lines through the means indicate the 95% confidence range.

While the restriction of pyrethroid to only the legs reduces the amount of insecticide needed per application, it can require applications to be made every 5–15 days, *i.e*, about twice as frequently as needed for whole body applications, if the efficacy against tsetse is to be maintained [15]. To elucidate the effect of treatment interval on dung beetles, the legs of oxen were treated just once at the start of 25-day assay periods, or at intervals of one and five days within these periods. The results of a study involving three replicates of each treatment regime (Fig. 5) showed, in accord with the data of Fig. 4, that the mortalities in the first few weeks after a single application were very low. They were hardly greater with applications made at intervals of five days. However, daily applications caused mortalities to rise to very high levels of about 70% after 25 days, with no evidence that a plateau had been reached by then.

## Discussion

We studied the numbers and condition of dung beetles found in and beside artificial pats made from the dung produced by cattle in the first few weeks after treating the cattle with deltamethrin applied to various body regions as SpotOn pour-on or Decatix spray. The results confirmed previous findings [7–11] that the normal whole-body treatments of cattle can threaten dung fauna. Much of the dung contamination seemed to occur because insecticide in the anal area transferred directly to the dung. Other contamination appeared to result from the cattle licking themselves. This accords with the indication that topically applied ivermectin, used to control ectoparasites of cattle, can contaminate dung due to licking [26].

The use of sprays or dips, as alternatives to the pour-ons, can reduce substantially the threat to dung fauna. Hence, it is fortunate that Decatix and other sprays or dips are about as potent as SpotOn and other pour-ons against tsetse [12]. Moreover, the dips and sprays are potentially more economical since they are usually about a quarter of the cost of pour-ons. The problem is that, unlike pour-ons, the dips and sprays require dip tanks or spray races, and need plenty of water year round—prerequisites that are neither available nor affordable in many tsetse-infested areas. Within this scenario, the restriction of treatments to the legs or legs plus belly is exciting in several ways. First, it reduces yet further the insecticide demand and the risks to dung fauna. Second, it requires relatively little water and can be achieved by comparatively inexpensive leg-baths [19], small hand-sprayers [22,23] or perhaps brushes. Third, even if the frequency of restricted applications were increased a little, to ensure adequate control of tsetse [15], there would still be substantial savings in insecticide costs and no material increase in the threat to dung fauna. It is only the very frequent treatments, at intervals less than five days, that should be avoided. If it is policy to adopt whole body treatments, it would be beneficial to dung fauna if the insecticide were not put directly on the anal region and not applied in concentrated form to places where it can be readily licked. While the anal region is the preferred aggregation site for the *Amblyomma* genus of ticks, failure to treat that region does not allow a marked accumulation of ticks there, provided treatments are given to the legs and lower body on which ticks can spend several days before progressing to their preferred location [27,28].

The present study is the latest contribution to a long and instructive association between the improvement of the cost-effectiveness of tsetse control and the reduction of co-lateral damage. Fifty years ago the main control methods were poorly selective, involving extensive destruction of host animals and vegetation, and the wide application of organo-chlorine insecticides [29]. Since then the situation has improved steadily, especially with those control measures that relate to host-tsetse relationships. First it was shown that effective tsetse control could be obtained by eliminating just a few of the most preferred species of wild host [30]. Later studies [31] found that removal of only one such host, warthog, was ineffective, but the work raised more prominently the question of why tsetse exhibit host preferences. Answers to this led to a fuller understanding of the attractiveness of natural baits and the marked improvement of artificial ones [32]. The upshot was that insecticide-based control could be more cost-effective if the insecticide were deposited only on sparsely distributed baits [32], so reducing greatly the demand for insecticide and limiting the number and types of non-target organisms at risk. The development of very small artificial baits for some tsetse [33] has reduced further the insecticide usage, by about 80%—roughly the extent to which insecticide demand is reduced by restricting application to only certain parts of cattle [15]. Moreover, the fact that insecticide need be applied only to the older cattle [16] allows even more reduction in insecticide usage and helps to ensure that young stock remains exposed to ticks, so developing resistance to tick-borne diseases [18].

The general principle in this history is that the seemingly separate objectives of improved cost-effectiveness and reduced co-lateral risks can be addressed together by modifying an existing control measure to restrict the level and distribution of its interventions. In this endeavour a close co-operation between ecologists and vector biologists can be particularly fruitful. With those insect pests showing strong responses to baits, a deserving field of study is the extent to which the selectivity of control can be improved by a fuller understanding of bait-orientated behaviour.

## Supporting Information

S1 Spreadsheet Data on which the figures are based. (XLS)
