# Correction: Pyrethroid Treatment of Cattle for Tsetse Control: Reducing Its Impact on Dung Fauna

**DOI:** 10.1371/journal.pntd.0003830

**Published:** 2015-06-19

**Authors:** The PLOS Neglected Tropical Diseases Staff

In the PDF download of this article, the publication date is listed incorrectly. The correct publication date is March 4, 2015.

The Funding section also contains an error. The Funding statement is repeated, appearing twice instead of just once. The correcting Funding statement is: Financial support was received from the DFID Animal Health Programme (Project nos. R7539, R7987), the DFID/RCUK programme on Zoonoses and Emerging Livestock Systems (ZELS, BBSRC grant no.BB/L019035/1), and the ICEF/UNDP/World Bank/WHO Special Programme for Research and Training in Tropical Diseases (TDR). The funders had no role in study design, data collection and analyses, decision to publish, or preparation of the manuscript.
